# Multiple Myeloma Masquerading as Ovarian Carcinosarcoma Metastases: A Case Report and Review of the Approach to Multiple Myeloma Screening and Diagnosis

**DOI:** 10.1155/2018/3029650

**Published:** 2018-09-24

**Authors:** Robert Stuver, Alec Petersen, Thomas A. Guerrero-Garcia, Ursula Matulonis, Paul Richardson, Prabhsimranjot Singh

**Affiliations:** ^1^Beth Israel Deaconess Medical Center, Department of Internal Medicine, 330 Brookline Avenue, Boston, MA 02215, USA; ^2^Brigham and Women's Hospital, Department of Internal Medicine, 45 Francis Street, Boston, MA 02115, USA; ^3^Dana Farber Cancer Institute, 450 Brookline Avenue, Boston, MA 02215, USA

## Abstract

Multiple myeloma is the most common plasma cell dyscrasia and causes 2% of all cancer deaths in Western countries. Ovarian carcinosarcomas are very rare gynecological malignancies and account for only 1–2% of all ovarian tumors. In this case, we report a 67-year-old woman with known relapsed ovarian carcinosarcoma who presented with headache and neck pain. She was found to have new lytic lesions in the cranial and thoracic regions. While these lesions were assumed to be metastases, a diligent approach detected an M-spike on serum protein electrophoresis and a monoclonal gammopathy with immunoglobulin G lambda monoclonal immunoglobulin on immunofixation. A bone marrow biopsy confirmed the diagnosis of multiple myeloma. To our knowledge, this is the first ever reported case of concomitant multiple myeloma and ovarian carcinosarcoma. Our case highlights the utmost importance of a systematic approach to lytic lesions and emphasizes the need to consider secondary malignancies in the evaluation of possible metastases. We used the International Myeloma Working Group guidelines for screening and diagnosing multiple myeloma, and we provide a thorough review of this updated approach.

## 1. Introduction

Multiple myeloma is the most common plasma cell dyscrasia and causes 2% of all cancer deaths in Western countries. Ovarian carcinosarcomas are very rare gynecological malignancies and account for only 1–2% of all ovarian tumors.

## 2. Case Presentation

A 67-year-old female with a history of ovarian carcinosarcoma presented to the hospital with one week of headache and neck pain.

Her malignancy had been diagnosed one year prior to presentation after she had presented to her primary care physician with abdominal pain. Radiographic imaging at that time showed a large pelvic mass, and the patient subsequently underwent radical cytoreductive surgery which included total abdominal hysterectomy, bilateral salpingo-oophorectomy, and omentectomy. Pathology showed a focal left ovarian carcinosarcoma with metastases to the right ovary, omentum, and posterior cul-de-sac. The patient underwent six cycles of carboplatin and paclitaxel.

Eight months after completion of chemotherapy, the patient presented to her oncologist with new right pelvic pain. Pelvic imaging showed a new, deep right pelvic mass, and the patient underwent surgical resection which confirmed disease recurrence. The patient was set to begin localized radiation therapy and further chemotherapy when she developed headache and neck pain and presented to the hospital.

Upon current presentation, she noted an intractable bandlike headache and neck pain. Physical examination revealed normal vital signs, a normal mental status assessment, and a nonfocal neurological examination. She had restricted range of motion at the neck and midline point tenderness in the upper thoracic spine.

**Figure 1:**
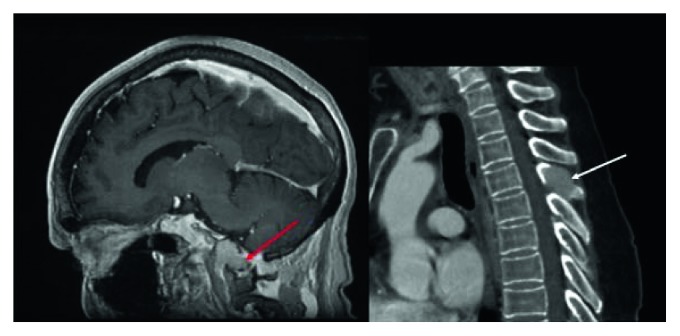
Magnetic resonance imaging (MRI) of the head and spine. MRI captured a 2.8 × 2.2 × 1.9-centimeter enhancing lytic mass centered in the left clivus and occipital condyle (red arrow). Additionally, an expansile soft tissue lesion was noted in the T4 spinous process (white arrow).

**Figure 2:**
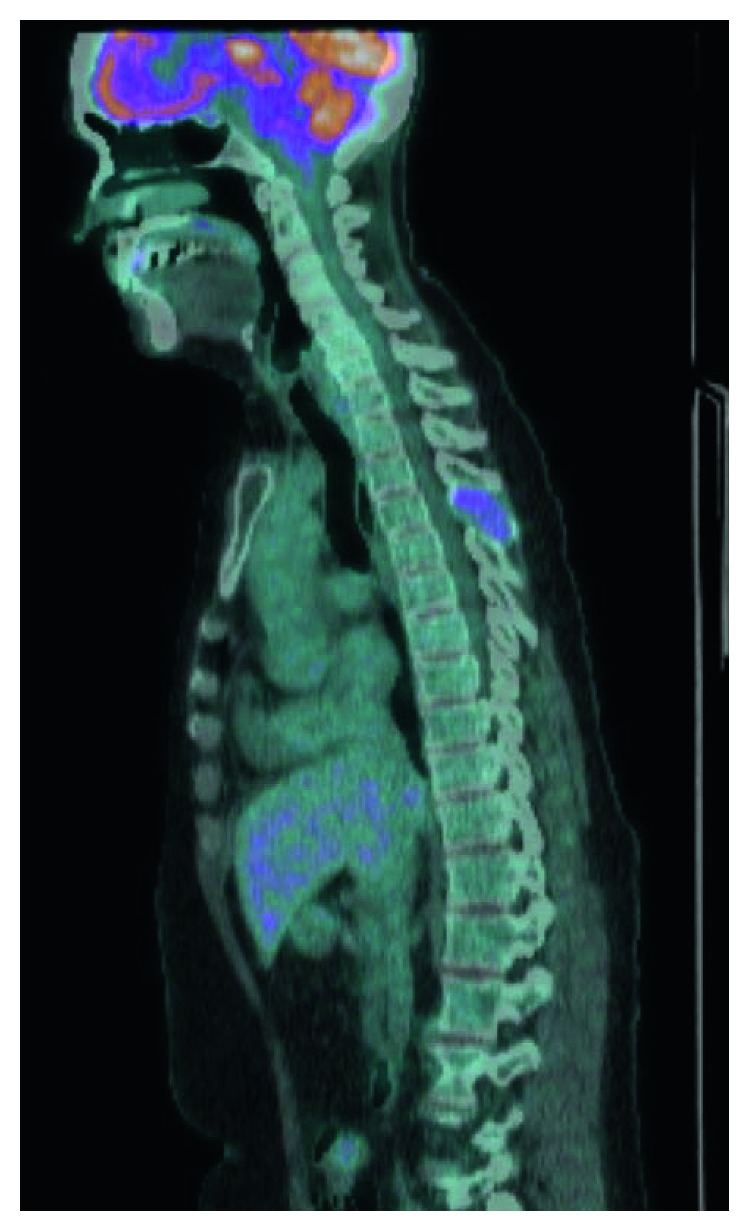
Positron emission tomography (PET). PET scan displayed a fluorodeoxyglucose (FDG)-avid lytic lesion in the left skull base where the previously noted mass on MRI was seen, as well as FDG uptake within the previously noted T4 lesion, seen here as the purple coloration within the T4 vertebral body.

Laboratory testing demonstrated a normal complete blood count, normal renal function, and normal serum electrolyte levels. Magnetic resonance imaging (MRI) of the head and spine were obtained and showed a lytic mass centered in the left clivus and occipital condyle, as well as an expansile soft tissue lesion in the T4 spinous process (Figure 1). A positron emission tomography-computed tomography (PET-CT) was also obtained (Figure 2). In the setting of known ovarian recurrence, these findings were assumed to be metastases.

**Figure 3:**
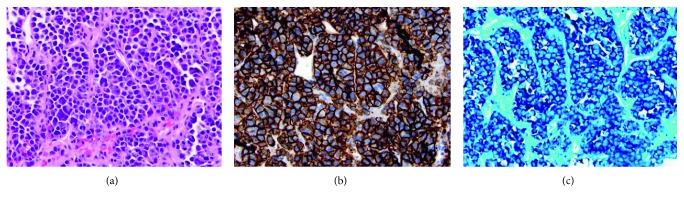
Bone marrow biopsy. (a) Staining with hematoxylin and eosin shows a population of abnormal plasma cells, many with binucleate and multinucleate forms. (b) Staining for CD138, a plasma marker, shows strong and diffuse positivity. (c) Fluorescence in situ hybridization (FISH) shows a monoclonal population with lambda light chain restriction, as essentially all cells are positive for lambda light chain and negative for kappa light chain.

However, a 1.83 g/dL M-spike (reference range: 0.80–1.70 g/dL) was detected on serum protein electrophoresis, and a monoclonal gammopathy with immunoglobulin G (IgG) lambda monoclonal immunoglobulin was seen on immunofixation. Lambda free light chains were elevated at 49.1 mg/L (reference range: 5.7–26.3 mg/L), and kappa free light chains were borderline decreased at 5.3 mg/L (reference range: 3.3–19.4 mg/L). The free kappa to free lambda ratio was abnormal at 0.12 (reference range: 0.26–1.65). No M-spike was detected on urine protein electrophoresis. A biopsy of the T4 lesion showed a plasma cell neoplasm, and a bone marrow biopsy showed a clonal population of >10%, confirming the diagnosis of multiple myeloma (Figure 3).

## 3. Discussion

This case is a startling demonstration of the coexistence of a solid and liquid malignancy, and we are unaware of any other reports in the literature of a concomitant presentation of metastatic ovarian carcinosarcoma and multiple myeloma. At first, the clivus and T4 lesions were presumed to be metastases; however, due to the uncommon location of these lesions for ovarian metastases, an alternative workup was pursued, and this diligent approach allowed for the correct diagnosis. This case highlights the importance of not overlooking the possibility of secondary malignancies. Additionally, our case emphasizes the correct approach to the diagnosis of multiple myeloma and provides a platform to review this topic.

Much has changed within the last decade in regard to the recommended screening tests and formal diagnosis of multiple myeloma, and a succinct review is necessary. The preferred screening test for multiple myeloma has become the combination of serum protein electrophoresis (SPEP) and immunofixation (SIFE) with serum free light chain analysis (FLC), as recommended by the International Myeloma Working Group (IMWG) in their consensus guideline [1]. These tests should be strongly considered in any patient with lytic bone lesions of indeterminate origin. These assays rely upon excess free light chains produced by proliferating neoplastic plasma cells and the subsequent disturbance in normal free light chain ratios. Importantly, the combination of SPEP and SIFE with FLC has replaced the need for urine studies in the initial screening for plasma cell dyscrasias and is a marked change in the historical tradition of screening with protein electrophoresis and immunofixation of the serum and urine. The most important study to evaluate these screening tests and accentuate this paradigm shift was performed by Katzmann et al. [2]. This group used the Mayo Clinic plasma cell database to identify 428 patients with positive urine IFE who also had SPEP, SIFE, and FLC in their diagnostic workup. SPEP and SIFE alone would have missed the diagnosis in 28 patients (6.5%); serum FLC alone would have missed the diagnosis in 60 patients (14%); however, SIFE with FLC together identified 99.5% of patients with positive urine studies. Hill et al. performed a similar prospective study of 923 patients and found that no substantial pathology would have been missed by replacing urine studies with FLC as an initial screen [3]. These studies, along with several others on the matter [4–6], have led the IMWG to state that the serum FLC in combination with SPEP and SIFE yields a great enough sensitivity to negate the need for urine studies when screening for plasma cell dyscrasias [1]. Moreover, FLC has prognostic implications and has become standard in assessing treatment response, so its measurement at onset cannot be understated. This approach likely has cost benefits as well, as Katzmann et al. note that based on available Medicare reimbursement at that time, serum FLC is $31 less than urine studies ($38 as compared to $71). An important caveat to this approach is if light-chain amyloidosis is suspected, in which case urine studies should still be obtained.

Like screening, the formal diagnosis of multiple myeloma has undergone updates within the last decade that deserve attention [7]. These updates, published in 2014 by the IMWG, are largely based on improved serum and radiographic diagnostics, such as the serum FLC assay, that allow for a diagnosis of multiple myeloma before the inevitable appearance of end-organ disease. Under these criteria, a diagnosis still can be made using the historic definition of a clonal population of bone marrow plasma cells ≥10% or a biopsy-proven boney or extramedullary plasmacytoma plus evidence of end-organ damage, which remains the classic CRAB criteria—hypercalcemia, renal insufficiency, anemia, or bony lesions on skeletal radiography, CT, or PET-CT. However, diagnosis has been updated to include a clonal population of bone marrow plasma cells or a biopsy-proven boney or extramedullary plasmacytoma plus any one of the following three biomarkers of malignancy: (1) clonal bone marrow plasma cell (BMPC) percentage ≥60%, (2) involved to uninvolved serum FLC ratio ≥100%, or (3) >1 focal lesion > 5 mm on MRI.

Earlier diagnoses using the aforementioned biomarkers of malignancy have allowed for earlier application of disease-altering therapy, and therefore clinicians must be aware of these novel definitions. These biomarkers identify patients who would have previously been classified as having smouldering multiple myeloma but have a great enough probability of progression to multiple myeloma that they deserve treatment. The IMWG previously stated that if a biomarker is associated with roughly an 80% probability of progression to multiple myeloma within two years, positivity should be regarded as a diagnosis of multiple myeloma [7]. The inclusion of BMPC ≥60% as a biomarker is largely based on a review by Rajkumar et al. of 651 patients who had been given a diagnosis of smouldering multiple myeloma [8]. They found that 21 patients (3%) had a BMPC ≥60%, and 95% of these patients progressed to multiple myeloma within two years. A similar finding was reported by Kastritis et al. [9]. The inclusion of involved to uninvolved serum FLC ratio ≥100% as a biomarker is largely based on a retrospective study by Larsen et al. [10] of 586 patients with a diagnosis of smouldering multiple myeloma. They found that an involved to uninvolved serum FLC ratio ≥100% predicted a 72% risk of progression to multiple myeloma within two years. When the involved FLC was also ≥1000 milligrams per liter (mg/L), the risk increased to 82%. This led the IMWG to state that when involved to uninvolved serum FLC ratio is ≥100%, the involved FLC must also be ≥1000 mg/L to be considered a true biomarker of malignancy. Finally, the inclusion of >1 focal lesion on MRI as a biomarker is based on 2 studies that showed that more than 1 focal MRI lesion was a strong predictor of progression to multiple myeloma within 2 years [11, 12]. Taken together, these biomarkers of malignancy are landmark additions to the definition of multiple myeloma. In our patient, criteria were met by having a clonal bone marrow population of ≥10% and >1 focal MRI lesions; importantly, as our patient had no overt evidence of end-organ disease, a reliance on the CRAB criteria could have delayed diagnosis.

## 4. Discussion of Management

Management of this patient's dual malignancies presented a complex challenge. It was deemed that her metastatic ovarian carcinosarcoma demanded immediate therapy, and she therefore was initiated on carboplatin and pegylated liposomal doxorubicin. We knew that chemotherapy for her carcinosarcoma would also provide antimyeloma effects. She was also started on zoledronic acid every 4 weeks. The patient has since completed six cycles and has no radiographic evidence of recurrent ovarian malignancy on CT scanning. Her clivus lesion remains stable in size, her T4 lesion has decreased in size, and her IgG lambda has decreased greater than 50%, equating to a partial response. She will continue zoledronic acid with consideration for bortezomib-based therapy if disease burden worsens.
